# Identification of the LCOR-PLCL1 pathway that restrains lipid accumulation and tumor progression in clear cell renal cell carcinoma

**DOI:** 10.7150/ijbs.107981

**Published:** 2025-02-26

**Authors:** Wen Li, Jian Shi, Qingyang Lv, Daojia Miao, Diaoyi Tan, Xiaojun Lu, Hairong Xiong, Qianqian Luo, Yaru Xia, Yuqi Han, Xuejiao Dong, Guixiao Huang, Xiaoping Zhang, Hongmei Yang

**Affiliations:** 1 Department of Pathogenic Biology, School of Basic Medicine, Tongji Medical College, Huazhong University of Science and Technology, Wuhan, China.; 2 Department of Urology, Union Hospital, Tongji Medical College, Huazhong University of Science and Technology, Wuhan, China.; 3 Institute of Urology, Tongji Medical College, Huazhong University of Science and Technology, Wuhan, China.; 4 College of Life Science and Technology, Hubei Key Laboratory of Quality Control of Characteristic Fruits and Vegetables, Hubei Engineering University, Xiaogan, China.; 5 Department of Urology, The Third Affiliated Hospital of Shenzhen University: Shenzhen Luohu Hospital Group Luohu People's Hospital, Shenzhen 518000, China.; 6 Shenzhen Huazhong University of Science and Technology Research Institute, Shenzhen, China.

**Keywords:** LCOR, ccRCC, PLCL1, lipid metabolism, apoptosis

## Abstract

Clear cell renal cell carcinoma (ccRCC) is the typical pathological subtype of renal cell carcinoma (RCC), representing about 80% of RCC. Reprogramming of lipid metabolism is one of the nonnegligible pathogeneses in ccRCC. Currently, the underlying regulatory mechanisms of lipid metabolism in ccRCC remain inadequately understood. In this study, we performed bioinformatics analyses and experiments both *in vivo* and *in vitro* to explore the biological functions and specific mechanisms of the ligand dependent nuclear receptor corepressor LCOR in ccRCC. Mechanistically, RUNX1 was a transcriptional suppressor of PLCL1, LCOR could interact with RUNX1 to relieve RUNX1-mediated repression of PLCL1, leading to increased PLCL1 expression, which, in turn, inhibited the tumor progression and lipid accumulation in ccRCC. Furthermore, PLCL1 decreased lipid accumulation through UCP1-mediated lipid browning and facilitated tumor apoptosis by activating p38 phosphorylation. In conclusion, the LCOR-RUNX1-PLCL1 axis provides a novel molecular mechanism underlying the progression and lipid storage of ccRCC. LCOR modulation represents a potential therapeutic strategy for the treatment in ccRCC.

## 1. Introduction

Renal cell carcinoma (RCC) is a prevalent malignant tumor in the urinary system and accounts for >90% of cancers in the kidney. Clear cell renal cell carcinoma (ccRCC) is the typical subtype of RCC, representing about 80% of RCC 1-3. At present, ccRCC has a poor prognosis due to the lack of effective clinical early diagnosis indicators, the resistance to targeted drugs, especially the first-line treatment drug sunitinib, and other factors 4,5. Therefore, it is urgent to unlock the ccRCC pathogenesis and find effective therapeutic target.

Metabolic reprogramming, an adaptive change made by tumors in response to rapid proliferation demands, is one of the most prominent features in breast cancer, prostate cancer, ccRCC and many other cancers 6-9. The most salient metabolic reprogramming in ccRCC is lipid metabolism dysregulation, leading to the abundant lipid storage in the cytoplasm 10,11. Therefore, ccRCC is considered to be a metabolic disease 9,12,13. There are lots of factors affecting lipid metabolism in ccRCC, one of which is lipid browning, a process that lipid is consumed through thermogenesis without producing adenosine triphosphate (ATP) energy 14. Uncoupling protein 1 (UCP1) can mediate lipid browning and accelerate lipid consumption. Moreover, our previous study has reported that phospholipase C-like 1 (PLCL1) reduced lipid accumulation and repressed ccRCC progression through lipid browning mediated via UCP1 15. Thus, abnormal lipid accumulation will promote the progression of tumors, playing a vital role in the pathogenesis of ccRCC 16,17. However, the mechanisms of lipid accumulation and consumption in ccRCC have not been fully clarified, hence the underlying mechanism of lipid metabolism in ccRCC requires to be further explored 18.

LCOR, a ligand dependent nuclear receptor corepressor, is recruited to agonist-bound nuclear receptors via a nuclear receptor (NR) box 19. LCOR could recruit C-terminal-binding proteins (CtBPs) or histone deacetylases (HDACs) to bind to transcription factors or nuclear receptors, exerting transcriptional inhibition 20-22. Interestingly, LCOR was also regarded as a transcription factor, driving transcriptional activation 23,24. Meanwhile, LCOR could regulate hepatic adipogenesis and repress the differentiation of 3T3-L1 adipocyte 25,26, involving in the regulation of lipid metabolism.

LCOR acts as a co-suppressor, binding various essential nuclear receptors in cancers, activating downstream signals and influencing the tumor progression. In breast cancer (BC), LCOR could physically interact with RIP140 to inhibit target gene expression induced by estrogen and decrease the proliferation of BC 27. In prostate cancer (PCa), LCOR interacted with KLF6 and then bound to the promoters of CDKN1A and CDH1, inhibiting KLF6 target genes transcription through recruiting CTBP1 and HDAC 20. LCOR could also repress the activation of androgen receptor (AR), restraining the growth of PCa 28. Low expression of LCOR in cervical cancer had a poor prognosis 29.

Mitogen-activated protein kinases (MAPKs) could influence multiple cellular activities associated with cancer 30. MAPKs activation facilitated the proliferation, migration and drug resistance of cancers 31-38. C-Jun NH2-terminal kinase (JNK), extracellular signal-regulated kinase (ERK) and p38 kinase (p38) are three major subfamilies in MAPKs 39. p38 is an important mediator of apoptosis by regulating related molecules such as caspases or Bcl-2 40,41. Activation of the ERK was involved in regulating the sensitivity of RCC to tyrosine kinase inhibitors (TKIs) or sunitinib 42-44.

Given LCOR exerts a pivotal efficacy in tumor progression and lipid metabolism, whereas its expression profile and functional significance in ccRCC remain unexplored, it necessitates to illustrate the role of LCOR in ccRCC. We conducted experiments to systematically evaluate the diagnostic potential and characterize its expression patterns. The assays both *in vitro* and *in vivo* were carried out to elucidate its underlying mechanistic involvement in ccRCC pathogenesis.

## 2. Materials and methods

### 2.1. Human ccRCC tissue and cell lines

Human ccRCC tissue and adjacent nonmalignant tissue were obtained from the Department of Urology, Union Hospital, Tongji Medical College, Huazhong University of Science and Technology (Wuhan, China) during 2022-2023. The Institutional Review Board of Huazhong University of Science and Technology had approved this study. The patients were diagnosed as ccRCC between February 2022 and February 2023, or underwent partial or total nephrectomy for ccRCC were included in this research. Patients with incomplete clinical information or two/more primary malignancies were excluded. All patients had written informed consent.

The HK2, A498, 786-O, CAKI, OSRC-2, HEK293T cell lines were bought from American Type Culture Collection (ATCC, USA). Cell completed culture medium was prepared with 500 mL DMEM (Gibco, MA, USA), 10% (50 mL) fetal bovine serum (FBS) and 1% (5mL) penicillin-streptomycin solution. The cells were cultured in a constant temperature incubator at 37°C.

### 2.2. RNA extraction and qPCR

Total RNA was extracted by the MagZol (#R4801, Magen, Guangzhou, Guangdong, China). HiScript II Q RT SuperMix (#R223-01, Vazyme, China) was used to reverse transcribe 1μg RNA into cDNA. SYBR qPCR Master Mix (#Q312-02, Vazyme, China), cDNA were used for qPCR assays by StepOnePlus™ PCR system (Applied Biosystems, California, USA). Samples were normalized by GAPDH. The calculation formula was 2^- ΔΔCT^. Primer sequences information was displayed in Supplementary Table 1.

### 2.3. Western blot

RIPA buffer (Beyotime, China), phenylmethylsulphonyl fluoride (PMSF) (#ST506, Beyotime), protease inhibitor (#P1005, Beyotime) were used to extract protein. Determined protein concentration via BCA protein assay kit (Beyotime, China). A total of 30μg denatured proteins were employed for gel electrophoresis with 10%-12% polyacrylamide gel. Finally, the ChemiDox XRS+ imaging system was used to detect protein expression. Antibodies information was displayed in Supplementary Table 2.

### 2.4. Immunohistochemistry (IHC)

Fixation the ccRCC tissue and adjacent nonmalignant tissue using 4% paraformaldehyde, then tissues followed by dehydration, paraffin embedding, sectioning, deparaffinization, and rehydration to extract antigens. Subsequently, the tissues were sealed with TBST containing 5% goat serum and incubated by primary antibody overnight at 4 °C. The next day, incubated tissues with the secondary antibody. Finally, stained tissues with DAB and hematoxylin (BS915, Biosharp, China).

### 2.5. RNA-sequencing analysis

Cellular RNA extraction and transcriptome sequencing were carried out by Majorbio (China).

### 2.6. CCK8 cell viability assays

Inoculated cells in 96-well plates with 2000 cells per well. Added 110μL CCK8 solution (CCK8, Yeasen) (CCK8: DMEM=1:10) to each well and incubated cells at 37°C. Measured the absorbance at 450 nm by spectrophotometer (NanoDrop Technologies, Wilmington, DE, USA).

### 2.7. Transwell assays

Collected and suspended 5×10^4^ A498 cells and 1.5×10^5^ CAKI cells after cells were starved for 2 days. Added 600ul completed medium to 24-well plate, put transwell ®inserts (01020023, Corning, USA) into the wells and added 250μL ccRCC cells suspension for migration experiments. For the invasion experiment, the number of cells was doubled, spread diluted matrix glue (354234, Corning, USA) (matrix glue: DMEM =1:9) into the insert in advance, and the rest was the same as in the migration experiment. After 24h for A498 and 48h for CAKI, the cells at the bottom of the insert were fixed with methanol and stained by crystal violet. The areas were photographed randomly by microscope.

### 2.8. Colony formation assays

LCOR overexpression and control cells were spread into 6-well plates at 1000 cells/well, then cultured cells 10-14 days approximately. Following this, washed cells with phosphate buffer saline (PBS), fixed and stained cells, observed the formation of colonies.

### 2.9. Establishment of cell lines

LCOR overexpression lentivirus (NM_032440), PLCL1 shRNA lentivirus (NM_006226), LCOR overexpression plasmid and METTL14 overexpression plasmid were bought from Genechem (shanghai, China). NR2F1 overexpression plasmid and RUNX1 overexpression plasmid were purchased from Genecreate (Wuhan, Hubei, China). LCOR siRNA and METTL14 siRNA were bought from GenePharma (shanghai, China). Lentivirus infection was performed according to the instructions of Genechem. Plasmid and siRNA transfection were conducted according to the instructions of lipo8000 (Beyotime, China).

### 2.10. Oil red staining

Fixed ccRCC cells by paraformaldehyde for 15 minutes, stained cells by oil red (#G1015, Servicebio) for 30 minutes. Images were captured by a microscope (#DSZ2000, UOP Photoelectric Technology).

### 2.11. Triglyceride (TG) and cholesterol (TC) detection

Inoculated cells in 6cm dishes and collected when the cells with high density, added 100μL 2% TritonX-100 (#P0096, Beyotime) to cells, then fully lysed cells for 30-40 minutes. The TG or TC content was determined by triglyceride assay kit (#A110-1-1), cholesterol assay kit (#A111-1-1, njjcbio, China), respectively.

### 2.12. Lipid fluorescence and lipid flow

Lipid fluorescence: LCOR overexpression plasmid, siRNA and corresponding controls were transfected into A498 and CAKI cells, respectively. After 48h, fixed cells and then incubated cells with 2% TritonX-100 for 5 minutes, subsequently, stained cells with BODIPY (493/503) (#HY-W090090, MedChemExpress) diluted 100-300 times for 30 minutes in the dark. Additionally, stained the nuclei with DAPI for 20 minutes. Fluorescence microscopy was used to detect green fluorescence at 488 nm and blue fluorescence at 405 nm.

Lipid flow: The procedures of plasmid and siRNA transfection were similar with lipid fluorescence. Stained cells with BODIPY (Diluted 50-100 times with PBS) for 30 minutes in the dark, digested cells with pancreatic enzymes, then collected and centrifuged cells, 250g for 5 minutes, washed cells with PBS and centrifuged again, resuspended cells in 300μL PBS. Finally, filtrated cell suspension with 35um filters and added them to the flow tube for flow cytometry.

### 2.13. TUNEL

Inoculated cells in 96-well plates with 7000 cells/well. After 24h, fixed cells, then incubated cells with PBS containing 0.3% TritonX-100 at room temperature for 5 minutes. Added TUNEL detection solution (#C10901, Beyotime) prepared according to the instruction to cells. Incubated cells at 37°C for 50 minutes. Finally, the fluorescence microscope was used to take pictures and the field of view was randomly selected.

### 2.14. Tube formation assay

Spread the 150μL diluted matrix glue (matrix glue: completed medium =2:1) to 48 well plates in advance. Collected ccRCC cells supernatant after cultivating 48h and centrifuged to purify the supernatant. Resuspended 1.5×10^5^ human umbilical vein endothelial cells (HUVECs) by 500μL supernatant and added them to 48-well plates spread with matrix glue previously, cultured cells at 37°C. After 4-8h, washed cells with PBS, added 500uL completed medium and 100uL diluted Cadmium-AM (#C1430, Thermo Fisher) (PBS: Cadmium-AM=100:1) to each well, then incubated cells at 37°C for 15 minutes. Fluorescence microscope was used to take pictures and the field of view was randomly selected.

### 2.15. Laser confocal

The A498 and CAKI cells were spread into confocal dishes with a density about 30%-40%. After 24-48h, fixed cells for 1h, then incubated cells with PBS containing 5%BSA + 5‰TritonX-100 for 15 minutes, PBS containing 5%BSA for 45 minutes, primary antibody (200μL) overnight at 4°C in the dark in turn. On the second day, cells were rewarmed for 90 minutes, then incubated cells by secondary antibody (200μL) for 90 minutes in the dark, after washing cells, added appropriate amount of anti-fluorescence quencher containing DAPI and incubated cells for 30 minutes. Images were photographed randomly with confocal laser microscope.

### 2.16. Nucleocytoplasmic separation assay

Cytoplasmic protein and nuclei protein were extracted by the cytoplasmic and nuclear protein extraction kit (#P0028, Beyotime).

### 2.17. Co-immunoprecipitation (Co-IP)

Incubated protein and primary antibody or IgG overnight at 4°C, remaining shaking. The next day, added protein A/G magnetic beads to the protein-antibody complex and shaken at 4°C for 4h. Washed beads with RIPA to remove the unbound proteins. The bound protein eluted from the beads were applied for western blot.

### 2.18. Chromatin immunoprecipitation (CHIP) assay

CHIP assays were carried out in A498 cells using the kit Simple CHIP® Kit (#9002, CST, USA) according to its instruction to verify the regulatory mechanism between NR2F1 and PLCL1, as well as RUNX1 and PLCL1. The main steps of CHIP assays were protein-DNA cross-linking, cell lysis, chromatin fragmentation, chromatin immunoprecipitation and qPCR. The designed primer sequences were as follows.

Full length: Forward 5'-GGAGCCTTAGGACCATGGTT-3'

Reverse 5'-GCAGGTGGGGAGTTTAGTCT-3'

Negative control: Forward 5'-TGACCTTCCCTAAATCCCCA-3'

(NR2F1) Reverse 5'-AACCATGGTCCTAAGGCTCC-3'

Negative control: Forward 5'-TCTGAAACCTCCCAAGTGCC-3'

(RUNX1) Reverse 5'-TTACGGTTGAGACAAAGGCC-3'

### 2.19. Dual luciferase reporter assays

Transfected specific plasmids into HEK293T cells and cultured cells at 37°C. After 48h, determined the luciferase activity via the dual luciferase assay kit (Promega, USA). The mutant and full-length plasmids of PLCL1 promoter region, the truncated and full-length plasmids of LCOR were purchased from Genecreate (Wuhan, Hubei, China), the construction vectors were pGL3-basic. Wild-type and 3'-UTR regions mutant plasmids of LCOR were bought from Tsingke (Beijing, China), the construction vectors were pmirGLO.

### 2.20. Animal model assays

Vital River Laboratories (Beijing, China) provided ageing 5 weeks male BALB/c nude mice, they were randomly divided 2 or 4 groups according to the arrangement of the experiment. In the subcutaneous tumor experiment, injected about 2×10^6^ CAKI cells into the nude mice armpits, the tumors volume and weight were measured every 3 days. After 4-5 weeks, the nude mice were killed by cervical dislocation and tumors were took out to measure the volume and weight. In the tumor metastasis experiment, injected about 1×10^6^ CAKI cells into the nude mice tail vein, about 8 weeks later, the tumor metastasis in nude mice was observed by live small animal fluorescent imaging assays using the Lago X system (Spectral instruments imaging, Tucson, AZ, USA). The Institutional Animal Use and Care Committee of Tongji Medical College had approved these animal studies.

### 2.21. Sunitinib sensitivity assays

The ccRCC cells were seeded in 96-well plates with 5000 cells/well. Next day, added sunitinib to cells at the concentration gradient of 0, 1, 2.5, 5, 7.5, 10, 20μM/μL. After 48h, added 110μL CCK8 solution (CCK8: DMEM=1:10) to each well and incubated cells at 37°C about 2-3h. Finally, measured the absorbance at 450 nm by spectrophotometer.

### 2.22. Bioinformatics analysis

Data on the bioinformatic analysis of LCOR was obtained from the cancer genome atlas (TCGA) (https://portal.gdc.cancer.gov). Pearson correlation coefficient analysis was utilized to identify the correlations between LCOR and PLCL1, PLCL1 and its transcription factors as well, P < 0.05 meant the data was significant. Gene Ontology (GO) analysis, Reactome annotations analysis, Gene set enrichment analysis (GSEA) and Kyoto encyclopedia of genes and genomes (KEGG) were applied to identify the functional enrichment of LCOR, as well as the signaling pathways that PLCL1 involved in. Used the JASPAR database (https://jaspar.elixir.no) to predict the PLCL1 transcription factors. The SRAMP database (http://www.cuilab.cn/sramp) was conducted to predict LCOR potential m6A modification site.

### 2.23. Statistical analysis

GraphPad prism 9.5 was utilized to analyze mean, SD or SEM and plot receiver operating characteristic (ROC) or area under the curve (AUC). Statistical analysis adopted t-test or analysis of variance with SPSS 22.0. The data was expressed as mean ± standard deviation. Experiments in this study were all carried out independently at least 3 times.

## 3. Results

### 3.1. LCOR was downregulated in ccRCC and related to a poor prognosis

**Figure 1:**
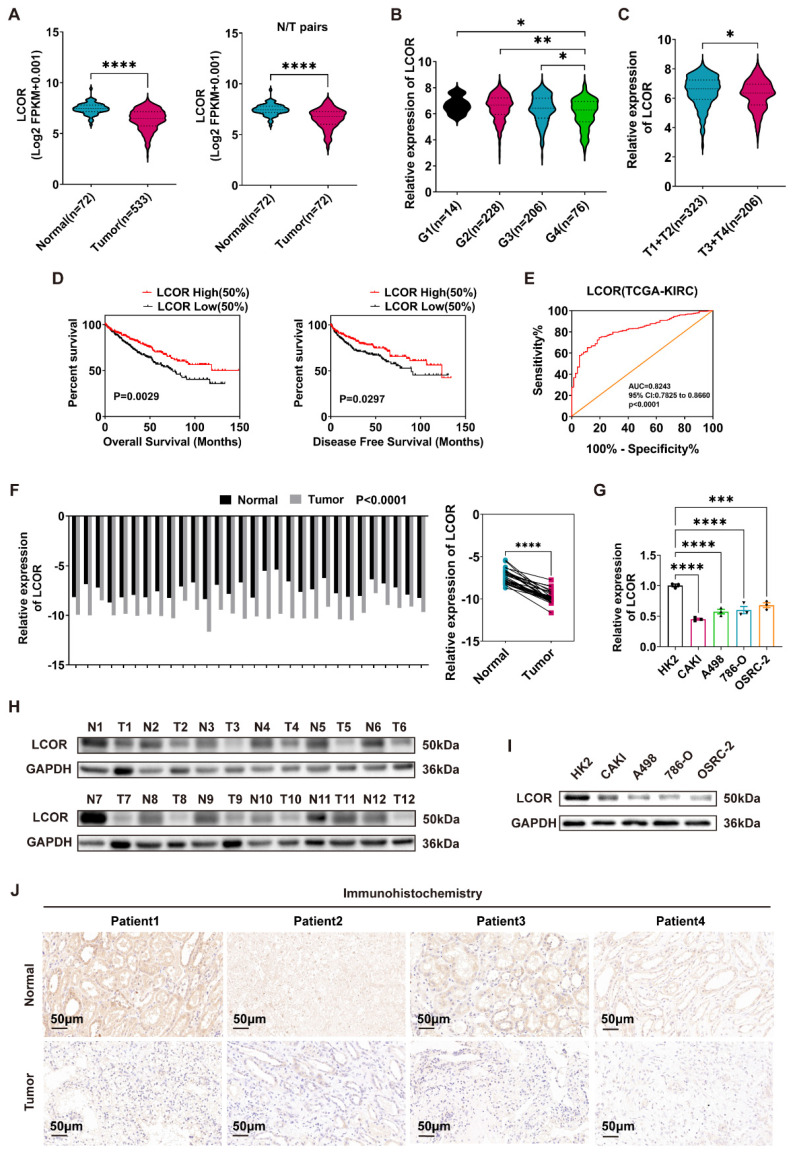
** LCOR was downregulated in ccRCC and related to a poor prognosis.** ****p < 0.0001, ***p < 0.001, **p < 0.01, *p < 0.05. **(A)** The mRNA levels of LCOR in 533 ccRCC tissue and 72 paired tissues in ccRCC based on data from the TCGA database. **(B)** The mRNA levels of LCOR in different G stages of ccRCC. **(C)** The mRNA levels of LCOR in different TNM stages of ccRCC. **(D)** The Kaplan-Meier curves of LCOR based on the TCGA database for both OS and DFS. **(E)** The ROC curve of LCOR based on the TCGA database (AUC = 0.8243; 95% CI: 0.7825 to 0.8660; p < 0.0001). **(F)** The mRNA levels of LCOR in 30 pairs of ccRCC tissue and adjacent nonmalignant tissue. p < 0.0001. **(G)** The mRNA levels of LCOR in 4 ccRCC cell lines (CAKI, A498, 786-O, OSRC-2) and control cell line (HK2).** (H)** The protein expression of LCOR in 12 pairs of ccRCC tissue and adjacent nonmalignant tissue. **(I)** The protein expression of LCOR in 4 ccRCC cell lines (CAKI, A498, 786-O, OSRC-2) and control cell line (HK2). **(J)** The IHC staining for LCOR in 4 pairs of ccRCC tissue and adjacent nonmalignant tissue.

Based on the critical role of LCOR in a variety of tumors, we analyzed the mRNA expression of LCOR in ccRCC based on TCGA-KIRC database. The results denoted that LCOR expression was obviously downregulated in ccRCC tissue (n=533) compared with normal tissue (n=72) and similar results were obtained from 72 paired matched cases (Fig. 1A). In addition, the levels of LCOR decreased with the increase of tumor histological grades (G stage) and tumor node metastasis degrees (TNM stage) (Fig. 1B, 1C). It suggested that downregulated LCOR indicated a poor prognosis in ccRCC patients. Kaplan-Meier survival curves were measured to explore whether survival time related to the LCOR expression. The results affirmed that the low levels of LCOR predicted a shorter survival time for overall survival (OS) and disease-free survival (DFS) (Fig. 1D). Receiver operating characteristic (ROC) curve analysis illuminated that LCOR had diagnostic value in ccRCC (Fig. 1E).

We further identified the LCOR expression in ccRCC tissue and cell lines. qPCR and western blot were conducted to illustrate that LCOR expression was lower in ccRCC cells (CAKI, A498, 786-O, OSRC-2) than that in control cell (HK2) on both mRNA and protein levels (Fig. 1G,1I). Similar results were observed in clinical patients' specimen (Fig. 1F,1H). The immunohistochemistry (IHC) staining assays also revealed that the expression of LCOR in ccRCC tissue was lower than that in normal tissue (Fig. 1J). In summary, LCOR is low expressed in ccRCC and relates to a poor prognosis.

In order to elucidate the phenomenon of LCOR downregulation in ccRCC, we screened METTL14 through bioinformatics analysis based on the fact that m6A is the most common RNA modification in mammals. qPCR results showed that METTL14 overexpression decreased LCOR mRNA levels in ccRCC cell lines. On the contrary, METTL14 knockdown increased mRNA levels of LCOR (Fig. S1A). To demonstrate the combination between METTL14 and LCOR, mutant LCOR 3'-UTR plasmid was constructed for the luciferase reporter assays (Fig. S1B, S1C). The results clarified that METTL14 knockdown inhibited LCOR expression through site 3003 in the 3'-UTR of LCOR (Fig. S1D). These findings uncover that METTL14-mediated m6A methylation modification decreases the LCOR expression in ccRCC.

### 3.2. LCOR repressed the progression of ccRCC *in vitro*

**Figure 2:**
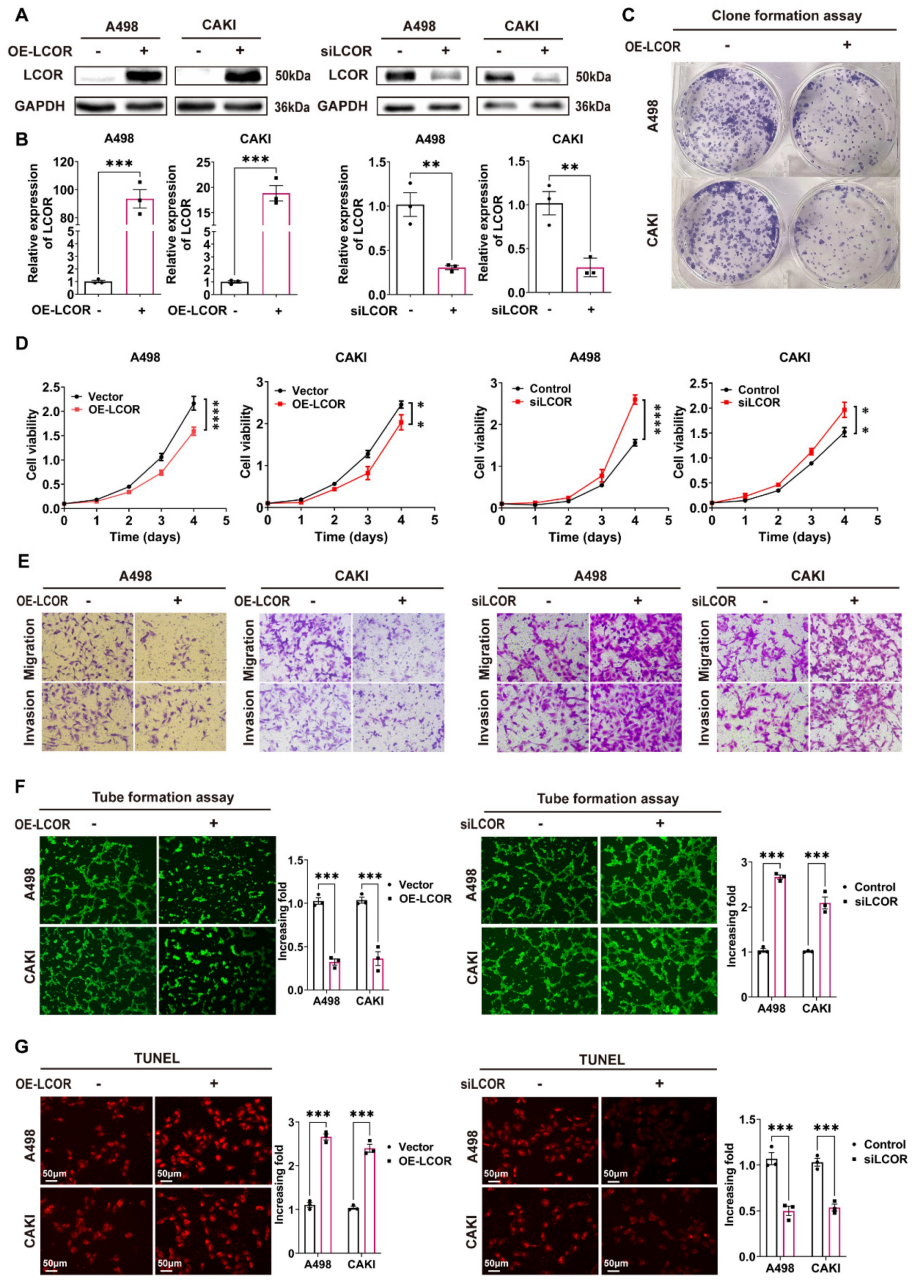
** LCOR repressed the progression of ccRCC *in vitro*.** There were at least 3 replicates in each independent experiment. ****p < 0.0001, ***p < 0.001, **p < 0.01. **(A) (B)** LCOR overexpression or LCOR knockdown ccRCC cell lines was established by infecting overexpression lentivirus or siRNA. Western blot and qPCR were used to identify the overexpression and knockdown of LCOR, respectively. **(C)** Colony formation assays of ccRCC cell lines with LCOR overexpression and control cells. **(D)** CCK8 assays of ccRCC cell lines with LCOR overexpression and knockdown to detect the proliferation abilities. **(E)** Transwell assays of ccRCC cell lines with LCOR overexpression and knockdown. **(F)** The effect of LCOR overexpression and knockdown of ccRCC cells lines on tube formation assays in HUVECs. **(G)** TUNEL fluorescence staining assays of ccRCC cell lines with LCOR overexpression and knockdown to detect the apoptosis levels.

To verify the biological functions of LCOR in ccRCC, we established ccRCC cell lines (A498 and CAKI) either with overexpression or knockdown of LCOR (Fig. 2A, 2B). Colony formation assays indicated that LCOR overexpression cells possessed the ability of decreasing proliferation (Fig. 2C). CCK8 assays showed that LCOR overexpression obviously inhibited ccRCC proliferation, whereas LCOR knockdown promoted cell proliferation (Fig. 2D). Transwell assays revealed that LCOR overexpression significantly impaired the migration and invasion abilities of ccRCC cells, whereas the depletion of LCOR improved these abilities (Fig. 2E, S2). Moreover, tube formation assays verified that the angiogenesis capacity of LCOR overexpression cells was attenuated and the capacity was enhanced in LCOR knockdown cells (Fig. 2F). TUNEL assays illustrated that the apoptosis level of LCOR overexpression cells was significantly elevated, whereas the LCOR knockdown cells decreased the apoptosis level (Fig. 2G). The above findings expound that LCOR has a tumor-suppressive effect in ccRCC.

### 3.3. LCOR suppressed lipid accumulation in ccRCC

**Figure 3:**
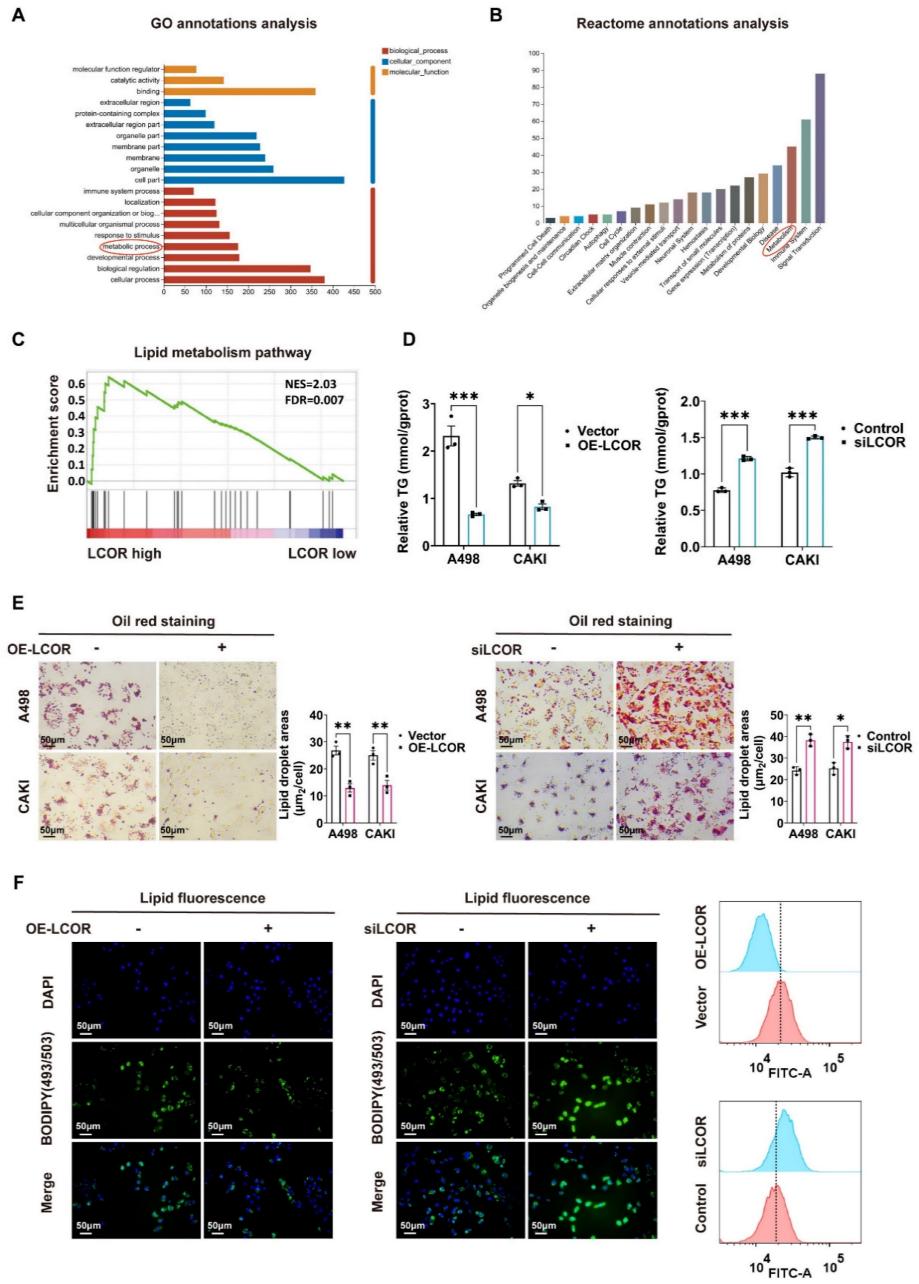
** LCOR suppressed lipid accumulation in ccRCC.** ***p < 0.001, **p < 0.01, *p < 0.05. The transcriptome sequencing (RNA-seq) was performed in A498 LCOR overexpression and control cells. **(A)** GO enrichment analysis of the RNA-seq. **(B)** Reactome annotations analysis of the RNA-seq. **(C)** The GSEA enrichment analysis of LCOR based on the TCGA database. FDR < 0.15 and p < 0.05 were statistically significant **(D)** The TG detection assays in ccRCC cell lines with LCOR overexpression and knockdown. **(E)** Oil red O staining assays and quantitative analyses of ccRCC cell lines with LCOR overexpression and knockdown. **(F)** Lipid fluorescence staining assays and quantitative analyses (Lipid Flow Cytometry) of A498 cells with LCOR overexpression and knockdown.

**Figure 4:**
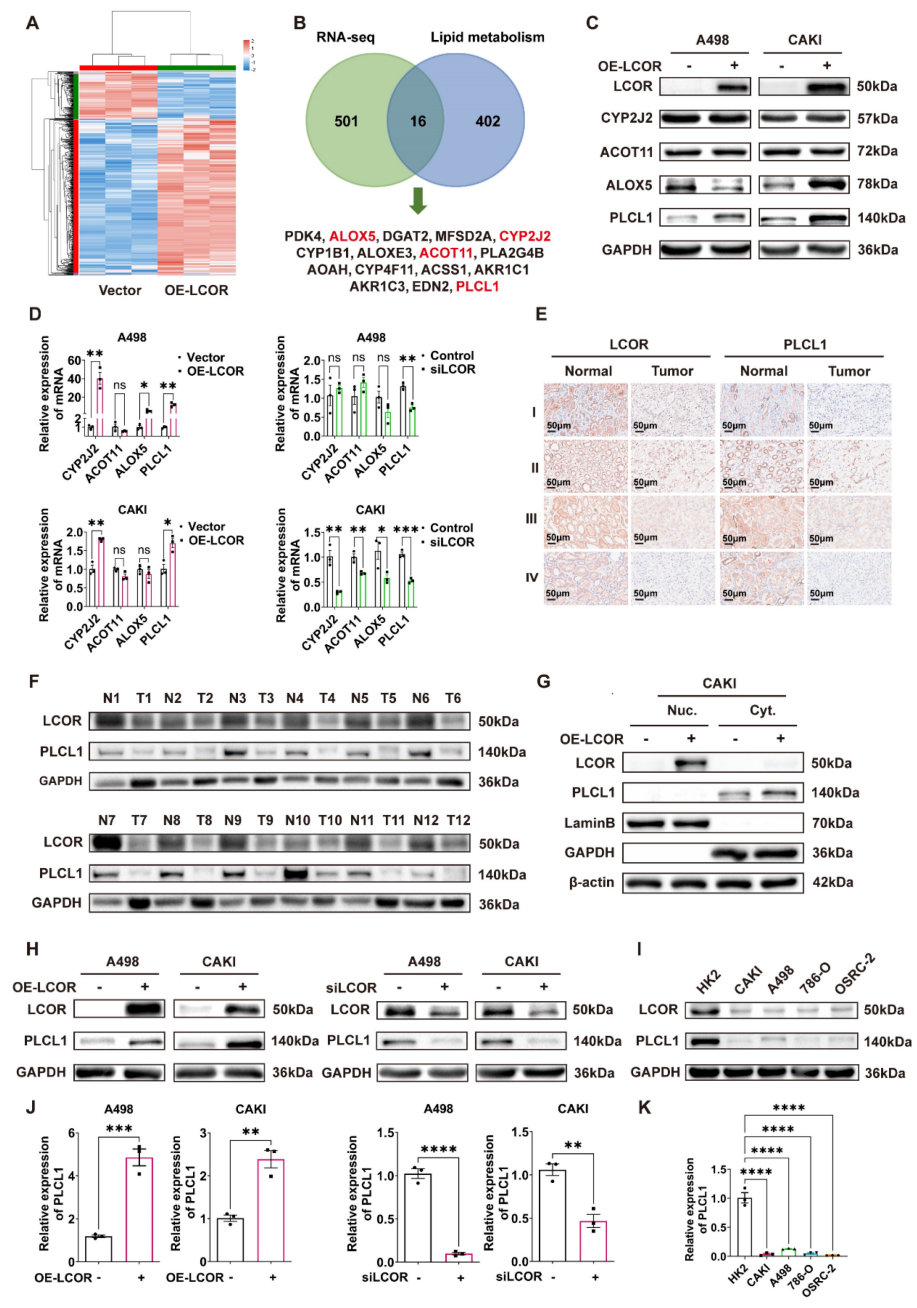
** LCOR positively regulated the expression of PLCL1.** ****p < 0.0001, ***p < 0.001, **p < 0.01, *p < 0.05. The transcriptome sequencing (RNA-seq) was performed in A498 LCOR overexpression and control cells. **(A)** The heatmap of cluster analysis based on RNA-seq. **(B)** The 16 genes were obtained from the intersection of RNA-seq and lipid metabolism gene sets enriched by GSEA. **(C)** The protein expression of CYP2J2, ACOT11, ALOX5 and PLCL1 in LCOR overexpressed ccRCC cell lines. **(D)** The mRNA levels of CYP2J2, ACOT11, ALOX5 and PLCL1 in LCOR overexpression and knockdown ccRCC cell lines. **(E)** IHC staining for LCOR and PLCL1 in 4 patients with different clinical grades of ccRCC tissue and adjacent nonmalignant tissue. **(F)** The protein expression of LCOR and PLCL1 in 12 pairs of ccRCC tissue and adjacent nonmalignant tissue. **(G)** Nucleocytoplasmic separation assay based on CAKI LCOR overexpression and control cells showed the distribution of LCOR and PLCL1. **(H)** Western blot was carried out to determine the protein expression of PLCL1 after LCOR overexpression and knockdown. **(I)** The protein expression of LCOR and PLCL1 in 4 ccRCC cell lines (CAKI, A498, 786-O, OSRC-2) and HK2 cell line. **(J)** qPCR was conducted to determine the mRNA levels of PLCL1 after LCOR overexpression and knockdown. **(K)** The mRNA levels of PLCL1 in 4 ccRCC cell lines (CAKI, A498, 786-O, OSRC-2) and HK2 cell line.

In view of the salient role of LCOR in the biological functions of ccRCC, we performed transcriptome sequencing (RNA-seq) in A498 LCOR overexpression and control cells (Fig. 4A). GO annotations analysis, Reactome annotations analysis and KEGG enrichment analysis of the RNA-seq affirmed that LCOR was associated with metabolism (Fig. 3A, 3B, S3A). GSEA enrichment analysis further verified that LCOR participated in lipid metabolism (Fig. 3C). It was found that LCOR overexpression cells possessed a lower triglyceride (TG) content, whereas the higher TG content was observed in LCOR knockdown cells (Fig. 3D). There was no significant difference in cholesterol (TC) content between LCOR overexpression and knockdown cells (Fig. S3B). Oil red staining results displayed that LCOR overexpression cells reduced lipid accumulation, whereas LCOR knockdown cells had the opposite function (Fig. 3E). Lipid fluorescence staining assays denoted that LCOR overexpression cells decreased lipid storage, LCOR knockdown cells obtained the more abundant lipid (Fig. 3F, S3C). These results support that LCOR can restrain ccRCC lipid accumulation.

### 3.4. LCOR positively regulated the expression of PLCL1

The differential genes from RNA-seq were intersected with the lipid metabolism gene sets analyzed by GSEA, and 16 genes were obtained (Fig. 4B). There were PDK4, ALOX5, DGAT2, MFSD2A, CYP2J2, CYP1B1, ALOXE3, ACOT11, PLA2G4B, AOAH, CYP4F11, ACSS1, AKR1C1, AKR1C3, EDN2 and PLCL1. Among these 16 genes, just ALOX5, CYP2J2, ACOT11 and PLCL1 had statistical differences in ccRCC tissue compared with normal tissue, the survival times of those genes had statistical significance as well (Fig. S4A, S4B). Other genes did not meet the requirements (Fig. S4C, S4D). Western blot was performed to identify the four genes expression in LCOR overexpressed ccRCC cell lines and results indicated that only PLCL1 was elevated in both A498 and CAKI cells, the ALOX5 was decreased in A498 but increased in CAKI cells, the CYP2J2 and ACOT11 remained unchanged (Fig. 4C). To further identify which gene was a downstream target of LCOR, qPCR was used to uncover that only PLCL1 possessed statistical significance in ccRCC cell lines with LCOR overexpression or knockdown (Fig. 4D).

Pearson correlation coefficient analysis revealed that LCOR was positively correlated with PLCL1 (Fig. S4E). IHC and western blot results showed that both LCOR and PLCL1 expressed lower in ccRCC tissue compared with normal tissue (Fig. 4E, S4F, 4F). Nucleocytoplasmic separation experiments found that LCOR was mainly expressed in the nuclei and PLCL1 was in the cytoplasm (Fig. 4G, S4G). In addition, western blot and qPCR were applied to further prove that LCOR positively regulated the expression of PLCL1. LCOR overexpression cells considerably improved the PLCL1 expression on both protein and mRNA levels, whereas the depletion of LCOR showed the opposite effect (Fig. 4H,4J). The protein expression of LCOR and PLCL1 were reduced in ccRCC cell lines (CAKI, A498, 786-O, OSRC-2) compared with those in HK2 cell line (Fig. 4I). The mRNA level of PLCL1 was also lower in ccRCC cell lines than that in HK2 cell line (Fig. 4K). These findings confirm that PLCL1 is a downstream gene of LCOR and is positively regulated by LCOR.

### 3.5. LCOR repressed ccRCC progression and lipid accumulation mainly through PLCL1

**Figure 5:**
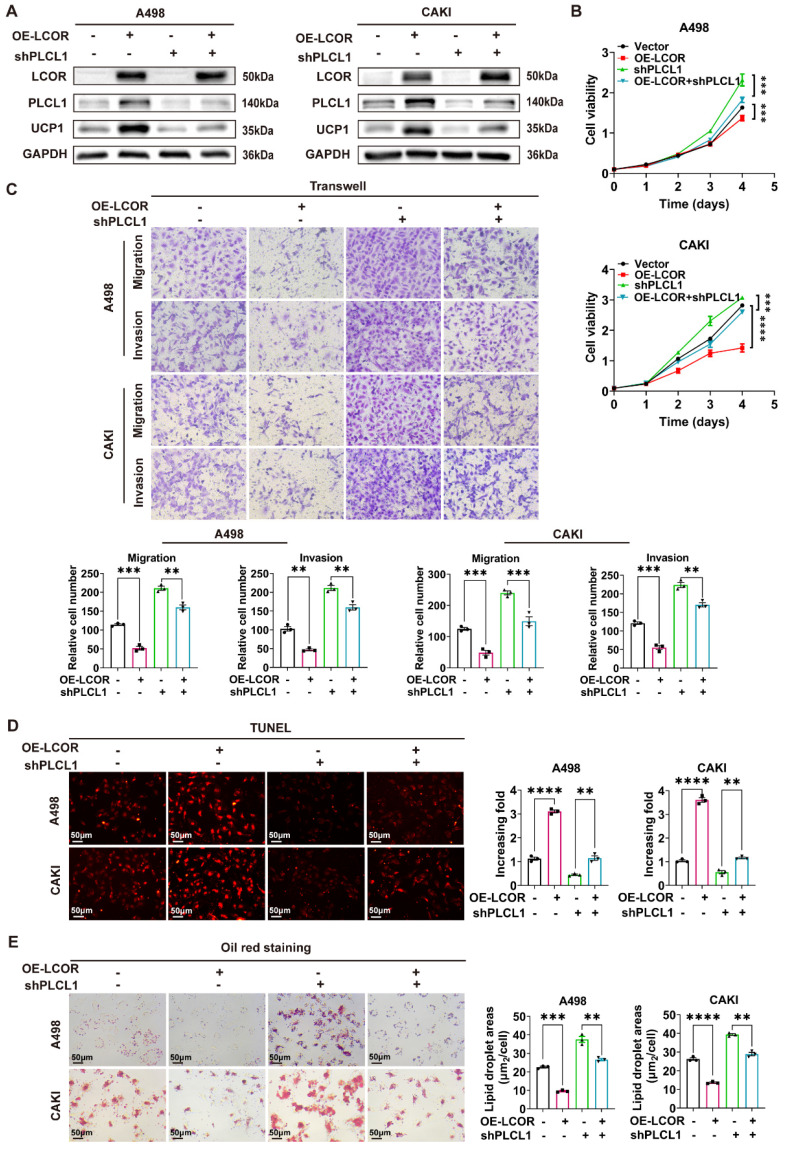
** LCOR repressed ccRCC progression and lipid accumulation mainly through PLCL1.** ****p < 0.0001, ***p < 0.001, **p < 0.01. PLCL1 shRNA lentivirus (shPLCL1) was used to establish functional compensation models in ccRCC cell lines with LCOR overexpression. The compensation experiments contained 4 groups: cell lines with LCOR overexpression control + shPLCL1 control, cell lines with LCOR overexpression lentivirus + shPLCL1 control, cell lines with LCOR overexpression control + shPLCL1, cell lines with LCOR overexpression lentivirus + shPLCL1. **(A)** The protein expression of LCOR, PLCL1 and UCP1 in functional compensation models. **(B)** CCK8 assays were performed in functional compensation models. **(C)** Transwell assays were conducted in functional compensation models. **(D)** TUNEL fluorescence staining assays were carried out to detect the apoptosis levels in functional compensation models. **(E)** Oil red O staining assays were applied in functional compensation models.

In order to illuminate the mechanism of LCOR regulating PLCL1 in ccRCC, functional compensation models were established in ccRCC cell lines with LCOR overexpression using PLCL1 shRNA lentivirus (Fig. 5A, S5A, S5B, S5C). UCP1 had been identified by our previous research to be a downstream gene of PLCL1 and mediated lipid browning 15. CCK8 assays indicated that the cell proliferation inhibition elicited by LCOR overexpression could be distinctly relieved by the knockdown of PLCL1 (Fig. 5B). Similarly, PLCL1 knockdown could observably reverse the inhibition of migration and invasion induced by LCOR overexpression in transwell assays (Fig. 5C). When it comes to apoptosis and lipid accumulation, similar results could also be observed, PLCL1 knockdown was able to alleviate the apoptosis increase and lipid accumulation decrease by LCOR overexpression (Fig. 5D, 5E). In short, the knockdown of PLCL1 could robustly relieve the biological efficacy induced by LCOR overexpression. Therefore, we conclude that LCOR suppresses the progression and lipid accumulation in ccRCC mainly through PLCL1.

To further investigate the PLCL1 involved signaling pathways, GSEA enrichment analysis was performed, along with a literature review, revealing that PLCL1 is closely associated with the WNT and MAPK pathways (Fig. S5D). The expression of key molecules in WNT (GSK-3β, β-catenin) and MAPK (JNK, p38, ERK) pathways were detected in ccRCC cell lines with LCOR overexpression by western blot, the results displayed that p-GSK-3β and p-β-catenin had no significant changes, p-JNK was unstable, p-ERK and p-p38 were obviously upregulated (Fig. S5E). The expression differences of molecules in MAPK pathway were more significant in LCOR overexpressed cells. It seemed unleashed that LCOR overexpression inhibited the malignancy of ccRCC, whereas MAPK activation promoted that in general. Therefore, we focused on the apoptosis-promoting function of p38.

We found that Bcl-2, an inhibitor of apoptosis, was downregulated in LCOR overexpressed cell lines (Fig. S5F), indicating that p38 exerted pro-apoptotic function rather than pro-tumor efficacy. To further verify the influence of p38 in ccRCC, the expression of p38 and Bcl-2 were detected in the functional compensation models by western blot. The results demonstrated that PLCL1 knockdown obviously reversed the upregulation of p-p38 and the downregulation of Bcl-2 caused by LCOR overexpression (Fig. S5G). These results unveil that PLCL1 participates in the regulation of apoptosis in ccRCC through the p38 pathway.

### 3.6. LCOR regulated the expression of PLCL1 by interacting with transcriptional suppressor RUNX1

**Figure 6:**
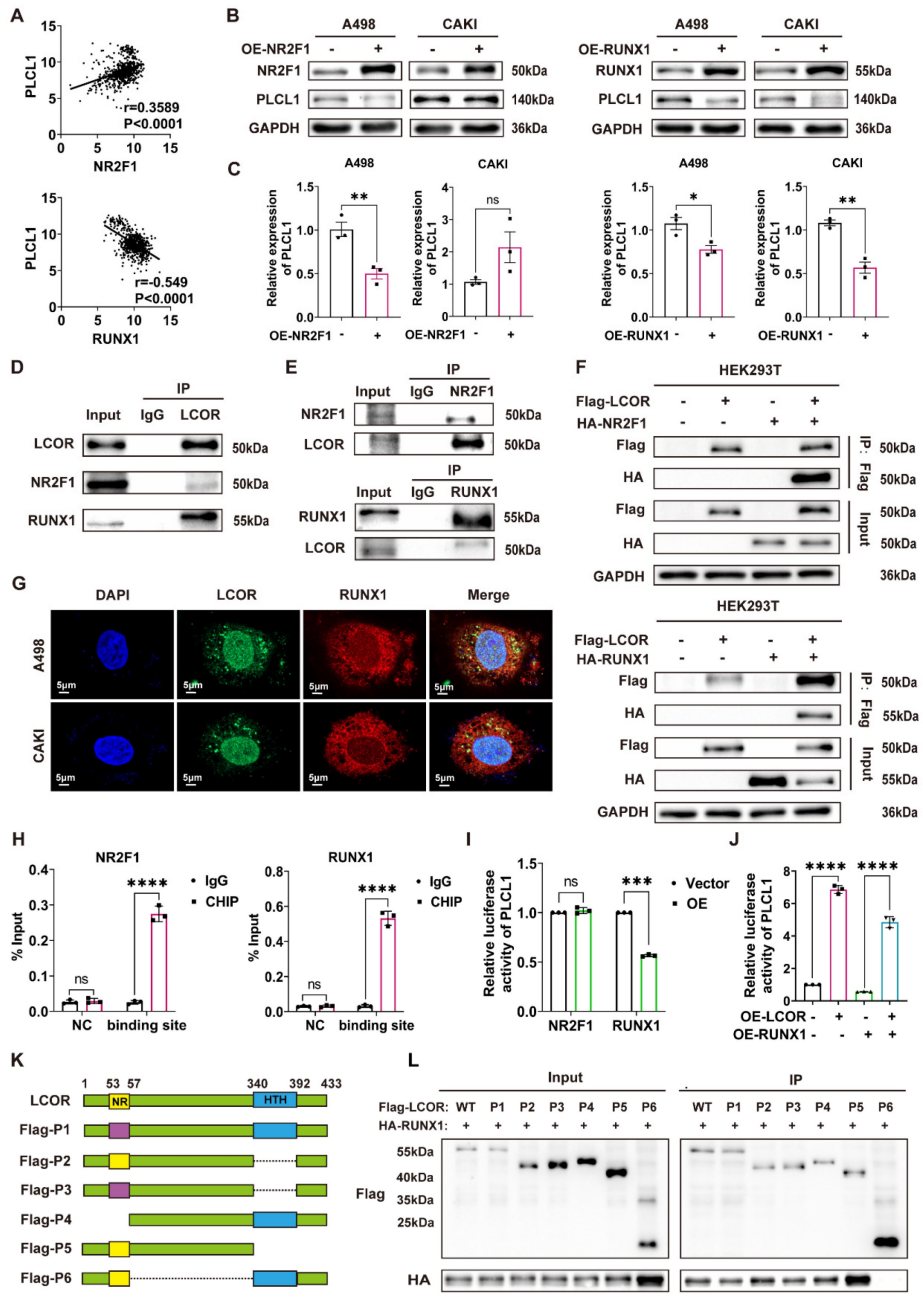
** LCOR regulated the expression of PLCL1 by interacting with transcriptional suppressor RUNX1.** ****p < 0.0001, ***p < 0.001, **p < 0.01, *p < 0.05.** (A)** The transcription factors (NR2F1, RUNX1) of PLCL1 predicted by JASPAR database. **(B) (C)** The protein and mRNA levels of PLCL1 in NR2F1 or RUNX1 overexpression cell lines, respectively. **(D)** The endogenous LCOR-NR2F1 or LCOR-RUNX1 interaction was determined through Co-IP assays in HEK293T. **(E)** The endogenous NR2F1-LCOR or RUNX1-LCOR interaction was determined via Co-IP assays in HEK293T. **(F)** The exogenous LCOR-NR2F1 or LCOR-RUNX1 interaction was determined via Co-IP assays in HEK293T. **(G)** Laser confocal assays were conducted to illustrate the co-localization of LCOR and RUNX1. **(H)** CHIP assays were applied in A498 cell line. **(I)** Dual luciferase assays were performed in HEK293T. **(J)** Dual luciferase assays were utilized in LCOR-RUNX1 functional compensation models. **(K)** The schematic drawing showed LCOR (full length, 1-433) and its six truncations. **(L)** Co-IP and western blot were carried out to verify the interaction fragment between RUNX1 and LCOR truncations.

Since LCOR could affect the mRNA and protein expression of PLCL1, we hypothesized that this occurred via transcriptional regulation. Five potential transcription factors of PLCL1 were obtained by JASPAR database. NR2F1 and RUNX1 had higher correlation with PLCL1 analyzed by Pearson correlation coefficient and were considered as the candidates (Fig. 6A, S6A). NR2F1 and RUNX1 overexpression models were established in ccRCC cell lines by transfecting overexpression plasmids, respectively (Fig. S6B, S6C). We found that NR2F1 and RUNX1 negatively regulated the PLCL1 expression on both protein and mRNA levels, except that there was no statistical difference in CAKI cells with NR2F1 overexpression (Fig. 6B, 6C). Therefore, both NR2F1 and RUNX1 might be transcription factors of PLCL1. However, bioinformatics analysis showed NR2F1 as a transcriptional activator of PLCL1, whereas the experimental results confirmed a negative regulatory effect.

Co-IP assays were carried out to identify the interactions between LCOR and NR2F1, LCOR and RUNX1 in A498, CAKI and HEK293T cells, respectively. Results exhibited that endogenous LCOR was efficiently immunoprecipitating NR2F1 or RUNX1, and endogenous NR2F1 or RUNX1 was also efficiently immunoprecipitating LCOR (Fig. 6D, S6D, 6E, S6E). To further elucidate these interactions, Flag-LCOR and HA-RUNX1 were co-transfected into HEK293T cells. The results uncovered that Flag-LCOR co-immunoprecipitated with HA-RUNX1, and Flag-LCOR also co-immunoprecipitated with HA-NR2F1 (Fig. 6F).

The results of laser confocal assays proved that LCOR and RUNX1 were mainly located in the nuclei (Fig. 6G). LCOR and NR2F1 obtained the similar results (Fig. S6F). Nucleocytoplasmic separation assays were applied in A498 and CAKI cell lines to further affirm the co-localization, it was found that LCOR and NR2F1 were mainly present in the nuclei, whereas RUNX1 was expressed in both nuclei and cytoplasm (Fig. S6G).

Chromatin immunoprecipitation (CHIP) assays expounded that NR2F1 or RUNX1 could directly combine with the promoter region of PLCL1, respectively (Fig. 6H). The dual luciferase reporter assays were conducted to demonstrate that NR2F1 had no effect on the transcriptional activity of PLCL1 promoter, whereas RUNX1 noticeably inhibited the transcription of PLCL1 (Fig. 6I). Based on this, we used LCOR overexpression plasmid to establish functional compensation models in HEK293T cells with RUNX1 overexpression, results of which showed that LCOR obviously reversed the transcriptional inhibition of PLCL1 and activated PLCL1 transcription (Fig. 6J). It suggested that RUNX1 was the main transcription factor during LCOR regulating PLCL1. In addition, RUNX1 could bind to the promoter of PLCL1 whether LCOR is overexpressed or knocked down (Fig. S6H, S6I). In order to define the precise site of PLCL1 promoter region binding to RUNX1, we predicted six possible binding sites by JASPAR database and constructed sequence mutations, obtaining six mutant plasmids, respectively (Fig. S6J). Transfected these plasmids into HEK293T cells and performed double luciferase assays, results of which illustrated that RUNX1 no longer inhibited the transcriptional activity of PLCL1 at site six (Fig. S6K). Therefore, site six was a potential site of PLCL1 combining with RUNX1. These data confirm that LCOR combines and alleviates RUNX1-repressed PLCL1 transcription, thereby increasing the expression of PLCL1.

To identify the binding domain of LCOR interacting with RUNX1, we constructed six Flag-tagged LCOR parts (Fig. 6K), these truncated plasmids were transfected into HEK293T cells. Among all the Flag-tagged LCOR parts, only Flag-P6 was unable to interact with RUNX1 (Fig. 6L), indicating that the domain from amino acids 57 to 340 was mandatory for the interaction with RUNX1. The Flag-P6 truncated plasmid (LCOR-N+C) was then transfected into A498 and CAKI cells, where it was observed that LCOR-N+C no longer elevated PLCL1 and UCP1 expression at either the protein or mRNA levels, in contrast to LCOR overexpression cells (Fig. S6L, S6M, S6N). These data confirm that the binding domain of LCOR interacting with RUNX1 is located between amino acids 57 to 340. We conclude that LCOR physically interacts with RUNX1 to regulate the expression of PLCL1.

### 3.7. LCOR inhibited ccRCC progression *in vivo*

**Figure 7:**
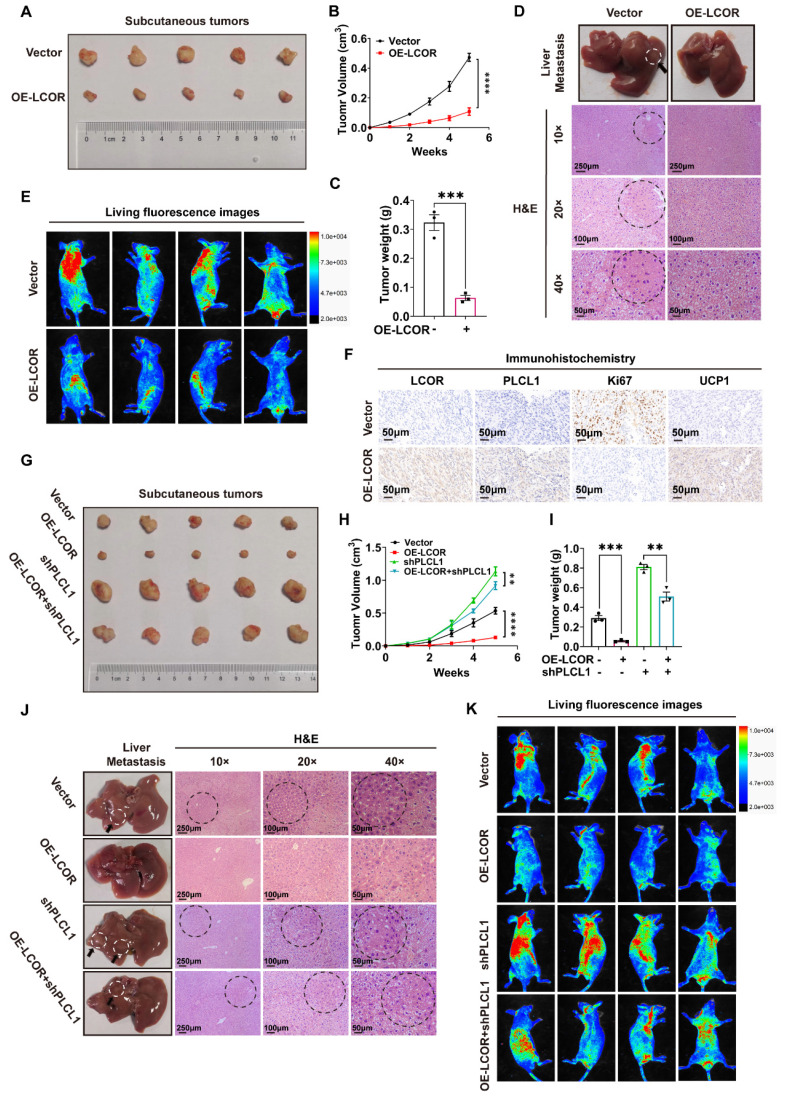
** LCOR inhibited ccRCC progression *in vivo*.** ****p < 0.0001, ***p < 0.001, **p < 0.01.** (A)** Images of tumors took out from LCOR overexpression and control groups. **(B)** Tumors volume was measured in LCOR overexpression and control groups.** (C)** Tumors weight was measured in LCOR overexpression and control groups. **(D)** H&E staining of the liver tissue from the metastasis models with or without LCOR overexpression. **(E)** Living fluorescence images of the metastasis models with or without LCOR overexpression. **(F)** IHC staining of LCOR, PLCL1, Ki67 and UCP1 in the subcutaneous tumors. **(G)** Functional compensation models contained 4 groups: vector, OE-LCOR, shPLCL1, OE-LCOR + shPLCL1. Images of tumors isolated from the functional compensation models. **(H)** Tumors volume was measured in the functional compensation models. **(I)** Tumors weight was measured in the functional compensation models. **(J)** H&E staining of the liver tissue from the functional compensation metastasis models. **(K)** Living fluorescence images of the functional compensation metastasis models.

On basis of the momentous effect of LCOR restraining ccRCC progression *in vitro*, we explored the function *in vivo*. Injected CAKI cells with LCOR overexpression or empty vector into BALB/c nude mice and monitored tumor volume and weight every three days. Subcutaneous tumors volume and weight took out from the mice with LCOR overexpression were considerably decreased compared with controls (Fig. 7A, 7B, 7C). H&E staining and live small animal imaging on basis of nude mice tail vein injection showed that LCOR overexpression obviously reduced the metastatic nodes number in the liver and repressed tumor metastasis (Fig. 7D, 7E). IHC results of subcutaneous tumors also unveiled that the expression of LCOR, PLCL1 and UCP1 increased and the tumor malignant index Ki67 decreased after LCOR overexpression (Fig. 7F). The above findings verify that LCOR suppresses ccRCC proliferation and metastasis *in vivo*.

To further confirm that PLCL1 exerted an essential role in the suppression of ccRCC by LCOR *in vivo*, PLCL1 shRNA lentivirus was used to establish functional compensation models in CAKI cells with or without LCOR overexpression. Consistent with *in vitro* experiments, PLCL1 knockdown significantly reversed the proliferation inhibition induced by LCOR overexpression (Fig. 7G, 7H, 7I). H&E staining and live small animal imaging based on compensation models also showed the similar results: PLCL1 knockdown reversed the metastasis inhibition caused by LCOR overexpression (Fig. 7J, 7K). In a word, PLCL1 is a crucial downstream gene by which LCOR inhibits the ccRCC progression.

In addition, sunitinib sensitivity assays displayed that LCOR overexpression noticeably increased the sensitivity of A498 and CAKI cells to sunitinib (Fig. S7A), whereas the high sensitivity of ccRCC to sunitinib elicited by LCOR overexpression could be obviously reversed by PLCL1 knockdown (Fig. S7B).

## 4. Discussion

In the current study we provided evidences that LCOR is inhibitory to ccRCC malignancy and lipid accumulation. Mechanically, LCOR co-binding relieves RUNX1-repressed PLCL1 transcription, leading to the upregulation of PLCL1 transcripts. PLCL1 then attenuates ccRCC lipid accumulation via motivating UCP1-mediated lipid browning and unleashes p38-dependent cell apoptosis pathways. In addition, LCOR overexpression would markedly sensitize ccRCC responsiveness to sunitinib, which is a standard first-line treatment for this disease. Taken together, we observed the LCOR-RUNX1-PLCL1 cascade accounts for multiple anti-tumor activities in ccRCC cells, upholding its candidacy as a novel diagnostic and therapeutic target.

Lipid accumulation is a critical driver in tumorigenesis, as exemplified by its protective effects on breast cancer and glioblastoma through defending the cytotoxicity 45. In ccRCC, lipid metabolism is reprogrammed to wire the oxidative decomposition and fatty acids synthesis pathways. Specifically, von Hippel-Lindau (VHL) inactivation would activate hypoxia-inducible factor (HIF) 46,47, which inhibits carnitine palmitoyl transferase 1A (CPT1A) gene transcription, mitigating lipid oxidative decomposition 48. Compromise in fatty acid synthesis would repress ccRCC tumor growth 49. Here we performed gene profiling and pathway enrichment analyses, uncovering the enrichment of LCOR signature in lipid metabolism. Notably, our previous work has confirmed that the LCOR target gene PLCL1 represses lipid accumulation in ccRCC by modulating UCP1-mediated lipid browning 15. Indeed, functional compensation tests in this report affirmed PLCL1 as a principal factor underlying LCOR-mediated lipid metabolism in ccRCC. Collectively, these findings position the LCOR-PLCL1 as a reprogrammed key module in ccRCC lipid metabolism and malignancy.

LCOR generally acts as a transcriptional co-repressor for nuclear receptors or other transcription factors 19-21,28, however, it can also function as a transcriptional activator to promote the expression of antigen processing/presentation mechanism (APM) gene 23 and certain ERα-induced genes 24. Here we found in ccRCC cells, LCOR reverses RUNX1-mediated repression of the PLCL1 gene. We do not know yet how exactly LCOR reverses the activity of RUNX1. Generally, LCOR would recruit co-factors (like CTBPs and HDACs) to the promoter of target genes 20,21,24 and regulate the interactions between nuclear receptor and co-activators 25. Nevertheless, our results showed that the affinity of LCOR-RUNX1 was mediated by the AA57-340 region, similar to LCOR interaction domain with HDAC6 20 that is distinct from the classical HTH domain 23,25,27.

The p38 pathway is one of three major MAPK pathways and its functionalities cover both tumor suppression and tumor promotion 50-52. Depending on cell type and stimuli, the p38 signaling also coordinate with other pathways to regulate mitochondrial activities and cell apoptosis 53,54. Consistent with our observations in ccRCC, p38 phosphorylation is linked with lipid metabolism 55,56 and UCP1-mediated browning of white adipose tissue 57. Importantly, here we demonstrated that LCOR overexpression considerably sensitizes ccRCC responsiveness to sunitinib, upholding an association between abnormal lipid metabolism and ccRCC sensitivity to sunitinib 58. In summary, these findings underscore the potential of targeting LCOR to synergize sunitinib efficacy in ccRCC.

## 5. Conclusion

Our study unveiled a potential molecular mechanism for the development of ccRCC, demonstrating that LCOR could interact with RUNX1 to relieve RUNX1-repressed PLCL1 transcription, leading to the upregulation of PLCL1 expression, which inhibited the tumor progression and lipid accumulation in ccRCC. This suggested that LCOR could be considered as a new clinical biomarker for the diagnosis and prognosis of ccRCC, offering an opportunity for the research of novel drugs targeting LCOR-RUNX1-PLCL1.

## Supplementary Material

Supplementary figures and tables.

## Abbreviations

RCC: Renal cell carcinoma
ccRCC: Clear cell renal cell carcinoma
LCOR: Ligand dependent nuclear receptor corepressor
UCP1: Uncoupling protein 1
ATP: Adenosine triphosphate
PLCL1: Phospholipase C-like 1
CTBPs: C-terminal-binding proteins
HDACs: Histone deacetylases
BC: Breast cancer
PCa: Prostate cancer
MAPKs: Mitogen-activated protein kinases
ERK: Extracellular signal-regulated kinase
JNK: C-Jun NH2-terminal kinase
p38: p38 kinase
TG: Triglyceride
TC: Cholesterol
CHIP: Chromatin immunoprecipitation assay
IHC: Immunohistochemistry
Co-IP: Co-immunoprecipitation
PBS: Phosphate buffer saline
qPCR: Quantitative real-time PCR
WB: Western blot
TUNEL: Terminal Deoxynucleotidyl Transferase mediated dUTP Nick-End Labeling
NR2F1: Nuclear receptor subfamily 2 group F member 1
RUNX1: RUNX family transcription factor 1
AR: Androgen receptor
PAX5: Paired box 5
TFAP2A: Transcription factor AP-2 alpha
VHL: Von Hippel-Lindau
HIF: Hypoxia-inducible factor
APM: Antigen processing/presentation mechanism
AA: Amino acid
HTH: Helix-turn-helix (DNA binding domain)
