# Supplementary material for: Identification of the LCOR-PLCL1 pathway that restrains lipid accumulation and tumor progression in clear cell renal cell carcinoma
Source: Int J Biol Sci. 2025 Feb 26;21(5):2296–312. doi: 10.7150/ijbs.107981 (PMC11900815; doi:10.7150/ijbs.107981)
Supplement: Supplementary file 1 — Supplementary figures and tables. [file ijbsv21p2296s1.pdf]

Supplementary figure 1

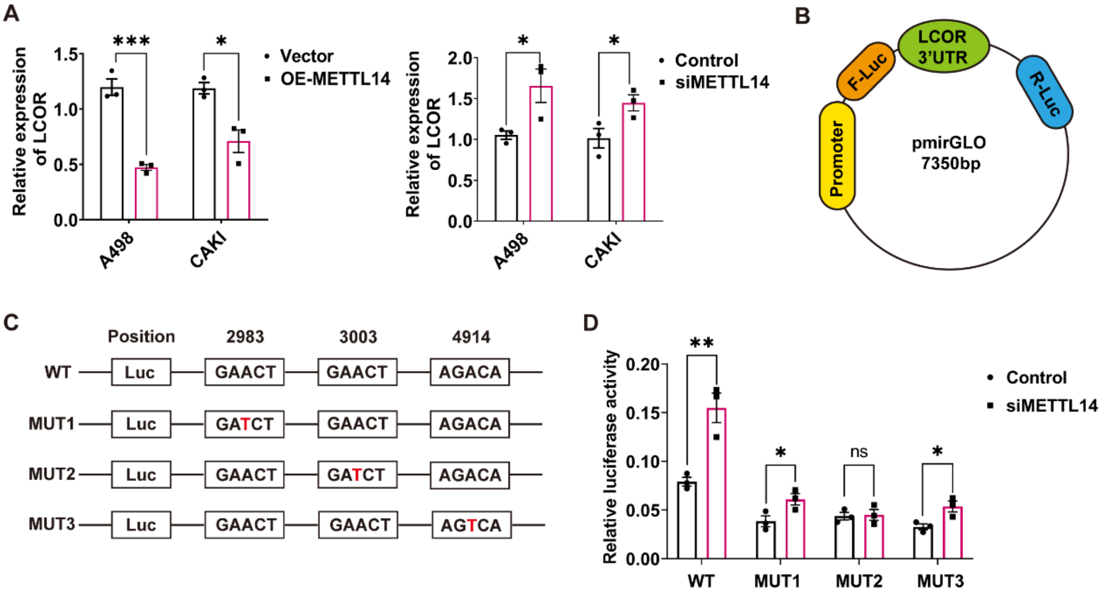

Supplementary figure 1. METTL14-mediated m6A modification was involved in LCOR downregulation. **(A)** The mRNA levels of LCOR in ccRCC cell lines with METTL14 overexpression and knockdown. **(B) (C)** A schematic diagram of a luciferase reporter gene that wild-type LCOR and mutant LCOR 3'-UTR at the m6A sequence sites were cloned into it. **(D)** Relative luciferase activity of the wild-type LCOR and mutant LCOR 3'-UTR reporter vectors in METTL14 knockdown HEK293T. ***$p < 0.001$, **$p < 0.01$, *$p < 0.05$.

Supplementary figure 2

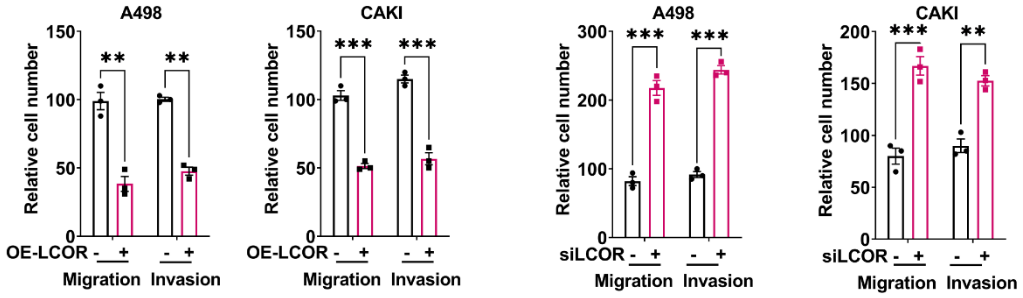

Supplementary figure 2. LCOR repressed the progression of ccRCC in vitro. The quantity analyses of transwell assays in ccRCC cell lines with LCOR overexpression or knockdown. ***p < 0.001, **p < 0.01.

Supplementary figure 3

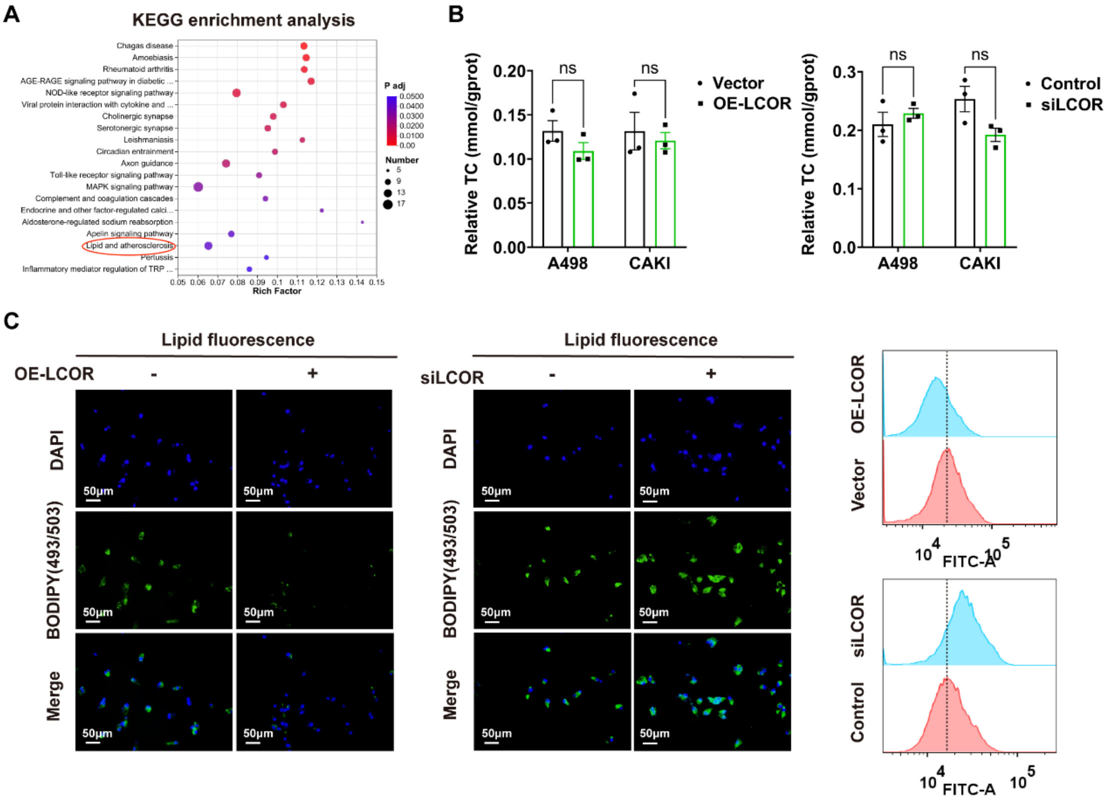

Supplementary figure 3. LCOR suppressed lipid accumulation in ccRCC. (A) KEGG enrichment analysis of the RNA-seq. **(B)** The TC detection assays in ccRCC cell lines with LCOR overexpression and knockdown. **(C)** Lipid fluorescence staining assays and quantitative analyses (Lipid Flow Cytometry) of CAKI cells with LCOR overexpression and knockdown.

Supplementary figure 4

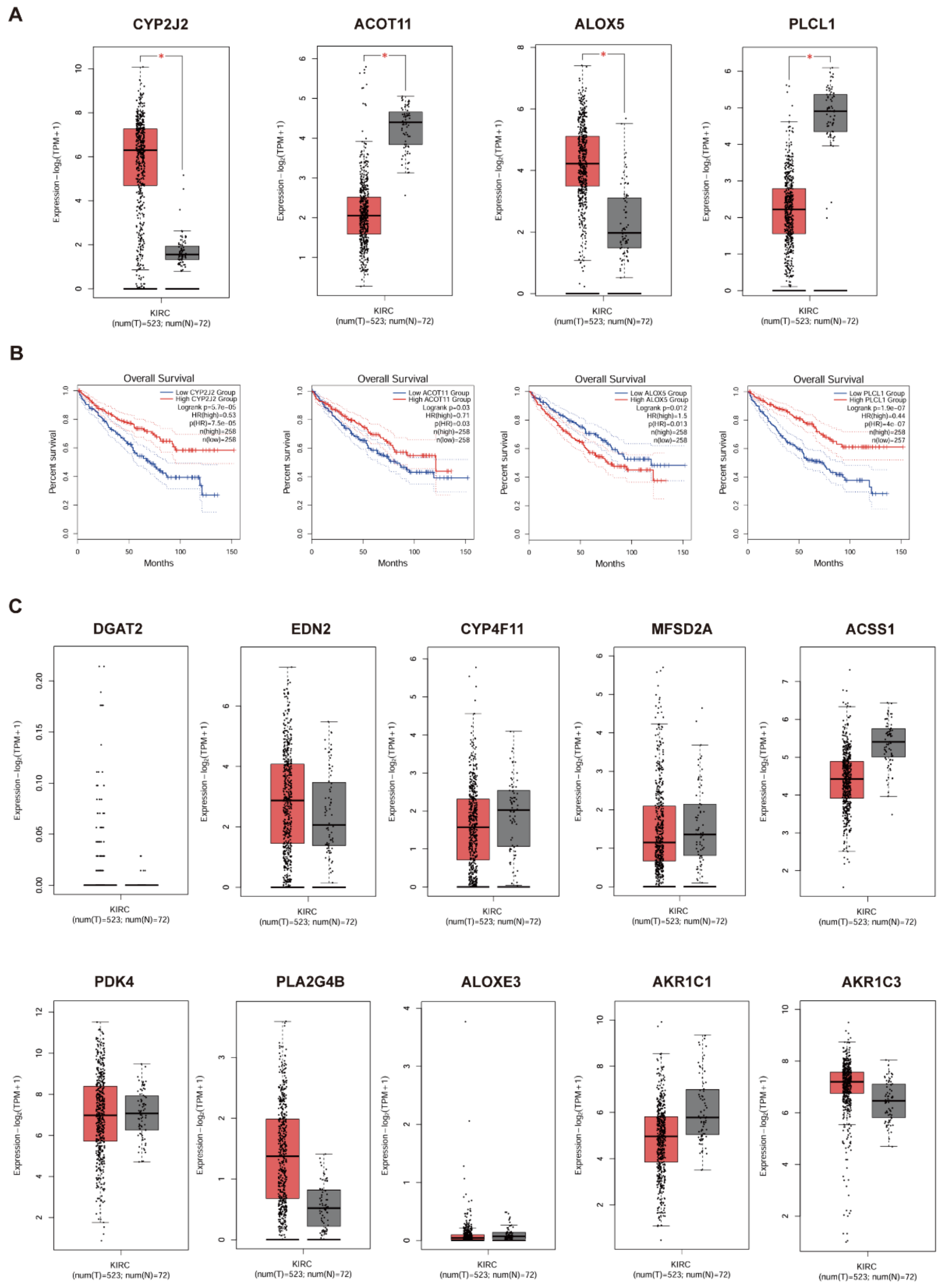

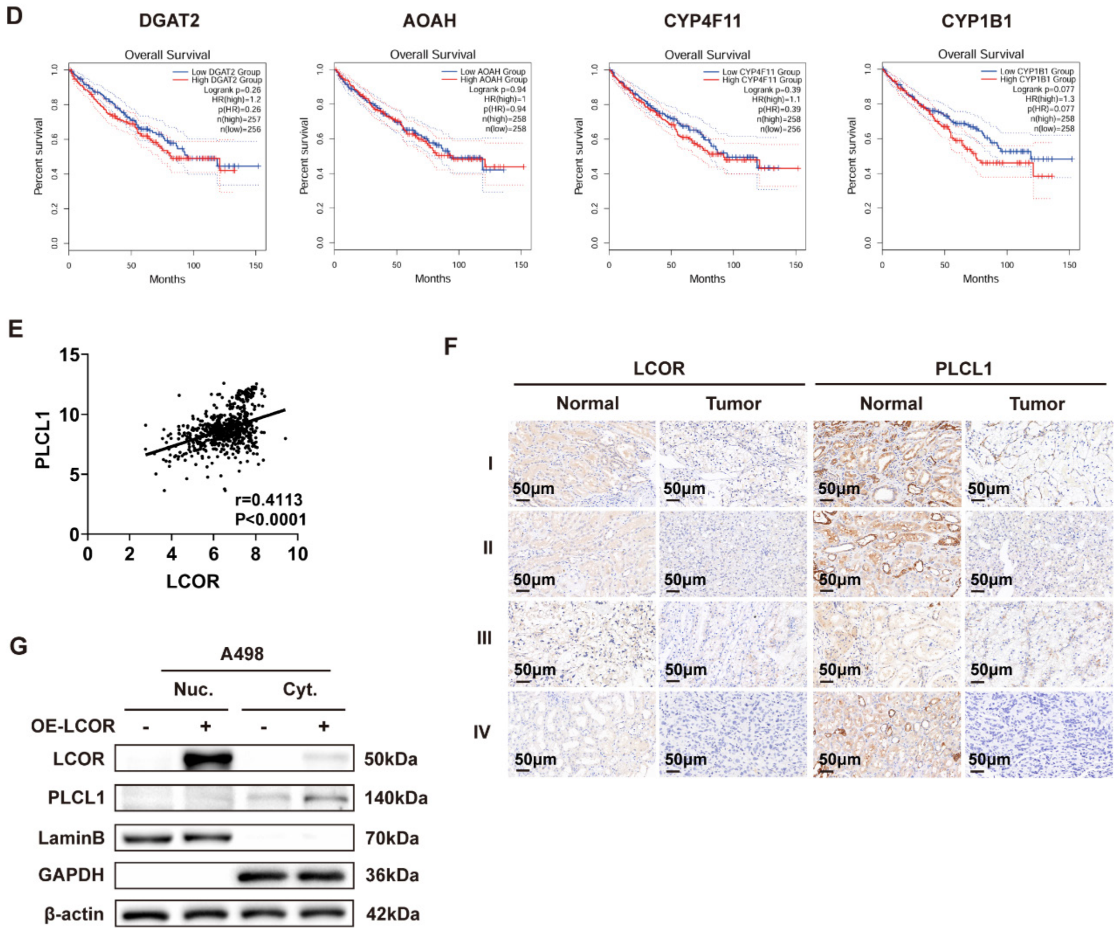

Supplementary figure 4. LCOR positively regulated the expression of PLCL1. (A) Analyses of significant differences between ccRCC tissue and adjacent nonmalignant tissue of CYP2J2, ACOT11, ALOX5 and PLCL1 using GEPIA2 database according to TCGA-KIRC project. **(B)** The Kaplan-Meier curves of CYP2J2, ACOT11, ALOX5 and PLCL1 based on the TCGA database for OS. **(C)** Analyses of significant differences between ccRCC tissue and adjacent nonmalignant tissue of DGAT2, EDN2, CYP4F11, MFSD2A, ACSS1, PDK4, PLA2G4B, ALOXE3, AKR1C1 and AKR1C3 using GEPIA2 database according to TCGA-KIRC project. None was statistically significant. **(D)** The Kaplan-Meier curves of DGAT2, AOAH, CYP4F11 and CYP1B1 based on the TCGA database for OS. None was statistically significant. **(E)** Pearson correlation

coefficient analysis was utilized to indicate the correlation between LCOR and PLCL1, $P < 0.05$ meant the data had statistical significance. **(F)** IHC staining for LCOR and PLCL1 in 4 patients with different clinical grades of ccRCC tissue and adjacent nonmalignant tissue. **(G)** Nucleocytoplasmic separation assay based on A498 LCOR overexpression and control cells showed the distribution of LCOR and PLCL1.

Supplementary figure 5

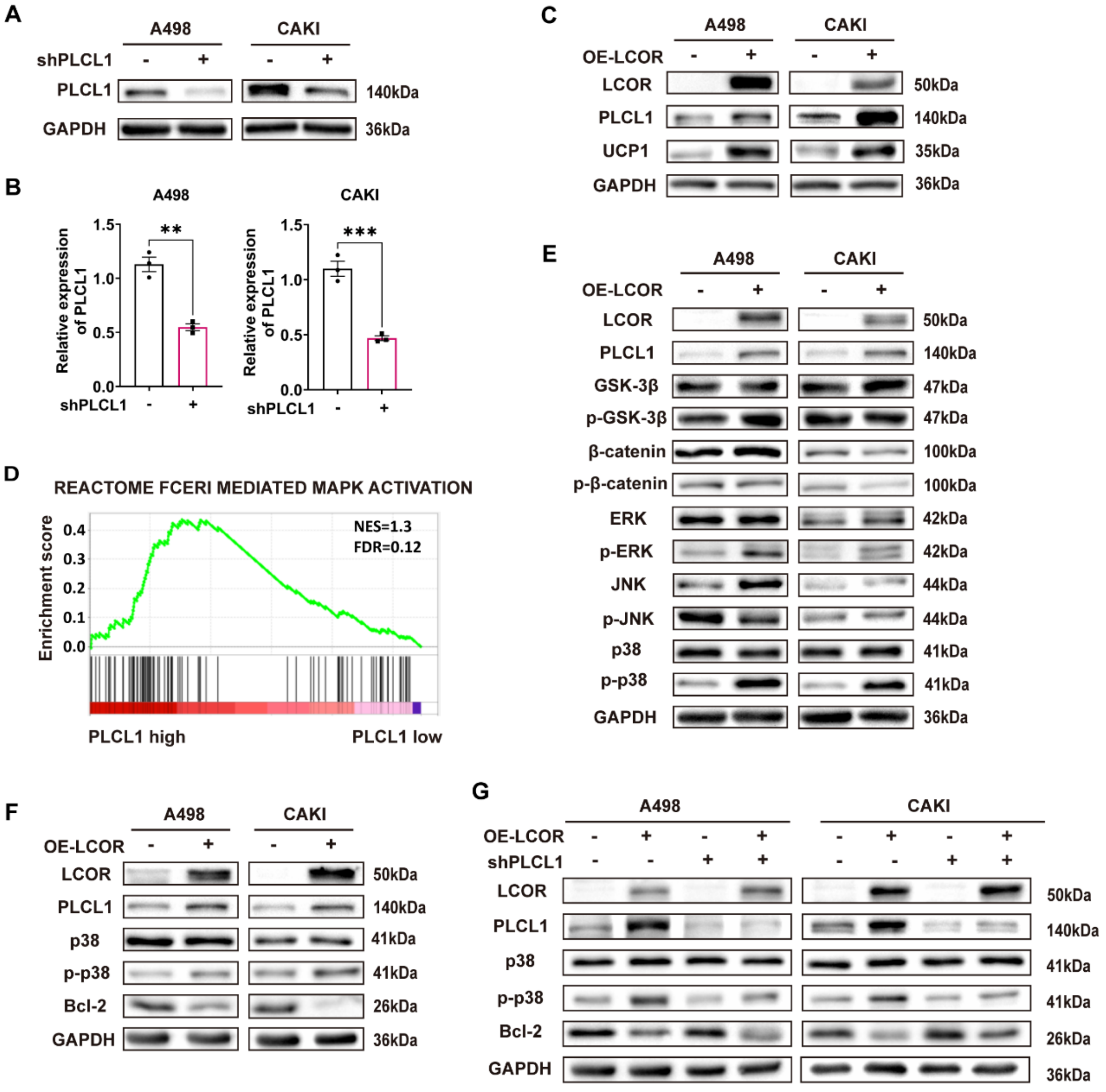

Supplementary figure 5. LCOR repressed ccRCC progression and lipid accumulation mainly through PLCL1. (A) (B) ccRCC cell lines with PLCL1 knockdown were established through infecting PLCL1 shRNA lentivirus. Western blot and qPCR were used to identify the knockdown of PLCL1, respectively. ***$p < 0.001$, **$p < 0.01$. **(C)** The protein expression of UCP1 in LCOR overexpressed cell lines. **(D)** The GSEA enrichment analysis of PLCL1 based on the TCGA database. FDR < 0.15 and $p < 0.05$ were statistically significant. **(E)** Western blot was applied in LCOR

overexpressed cell lines to detect the protein expression of key molecules in WNT and MAPK pathways. **(F)** Western blot was performed in LCOR overexpressed cell lines to detect the p38 and Bcl-2 protein expression. **(G)** Western blot was carried out in functional compensation models to detect the p38 and Bcl-2 protein expression.

Supplementary figure 6

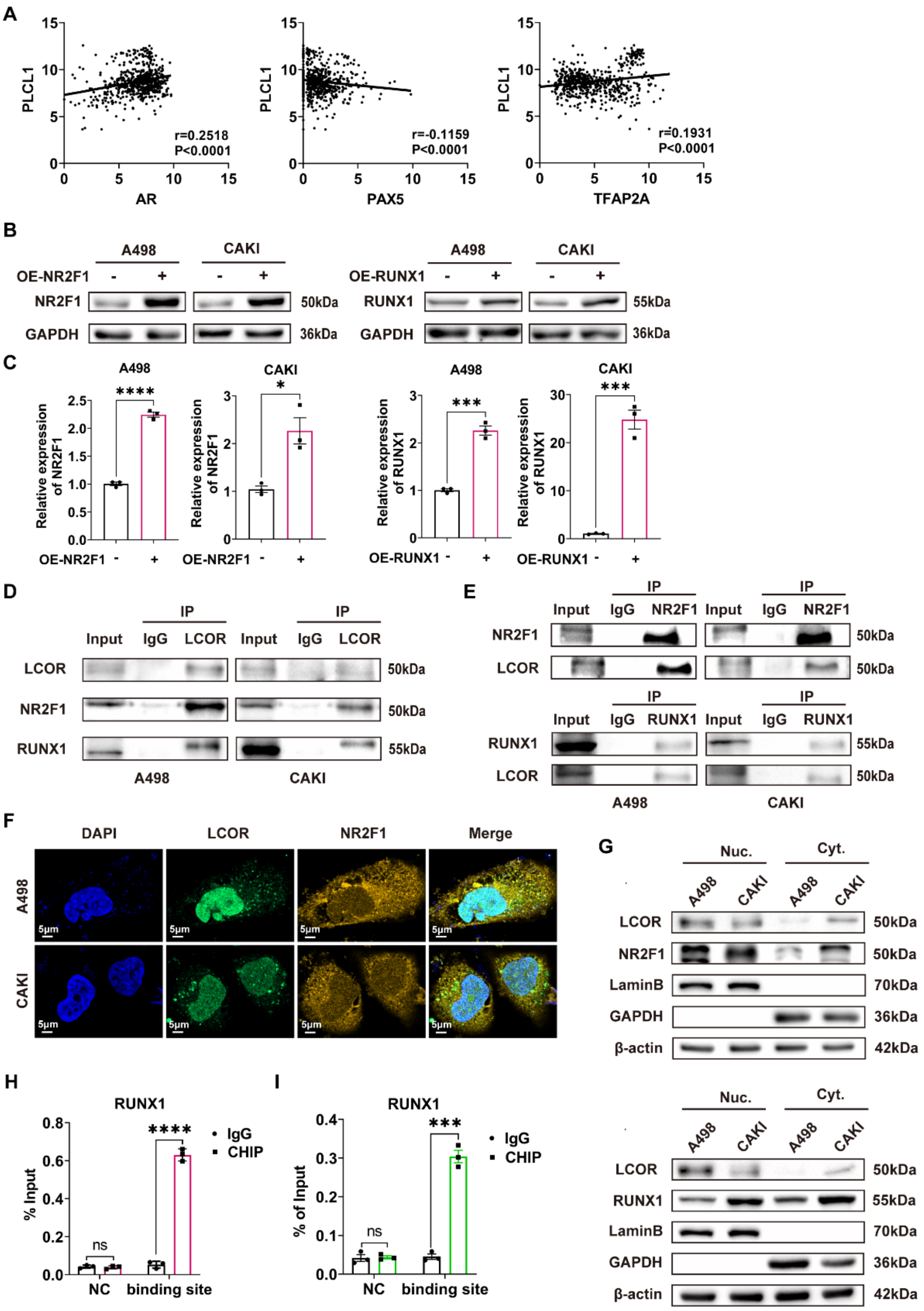

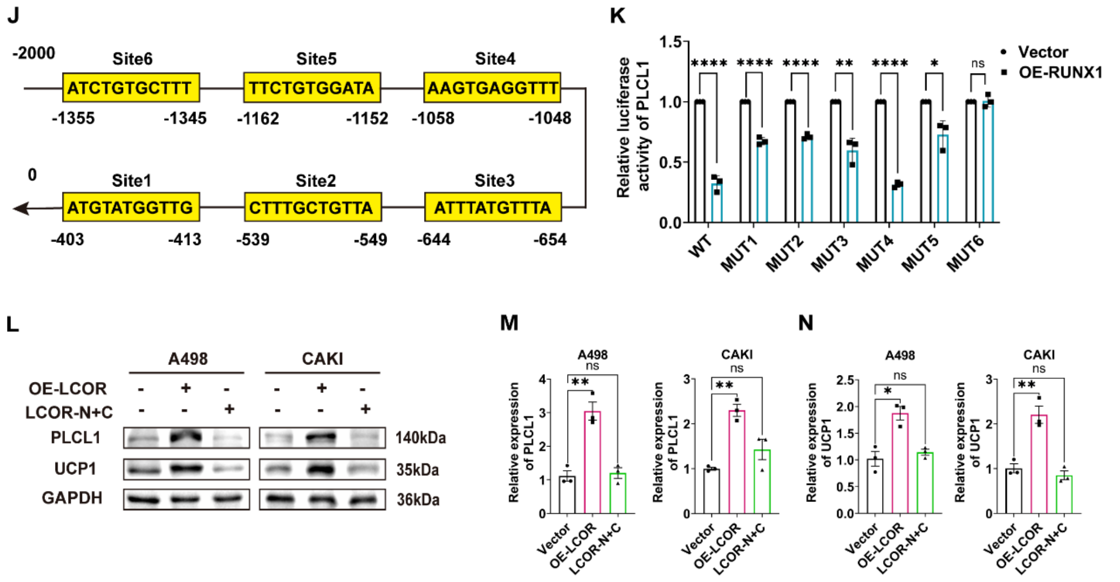

Supplementary figure 6. LCOR regulated the expression of PLCL1 by interacting with transcriptional suppressor RUNX1. ****$p < 0.0001$, ***$p < 0.001$, **$p < 0.01$, *$p < 0.05$. **(A)** The transcription factors (AR, PAX5, TFAP2A) of PLCL1 predicted by JASPAR database. **(B)** **(C)** NR2F1 overexpression or RUNX1 overexpression ccRCC cell lines was established via transfecting overexpression plasmids, respectively. Western blot and qPCR were conducted to identify the overexpression of NR2F1 or RUNX1. **(D)** The endogenous LCOR-NR2F1 or LCOR-RUNX1 interaction was detected through Co-IP assays in A498 and CAKI. **(E)** The endogenous NR2F1-LCOR or RUNX1-LCOR interaction was detected via Co-IP assays in A498 and CAKI. **(F)** Laser confocal assays were carried out to illustrate the co-localization of LCOR and NR2F1. **(G)** Nucleocytoplasmic separation assays based on A498 and CAKI cell lines showed the distribution of LCOR and NR2F1, as well as LCOR and RUNX1. **(H)** CHIP assays were applied in A498 LCOR overexpressed cell line. **(I)** CHIP assays were conducted in A498 LCOR siRNA knockdown cell line. **(J)** The diagram showed the 6 potential sites of PLCL1 promoter region binding to RUNX1 predicted by JASPAR

database. **(K)** Dual luciferase assays were applied to verify the precise site of PLCL1 promoter region binding to RUNX1. **(L) (M) (N**) Flag-P6 truncated plasmid (LCOR-N+C) was transfected into A498 and CAKI cells, western blot and qPCR were utilized to determine the expression of PLCL1 and UCP1.

Supplementary figure 7

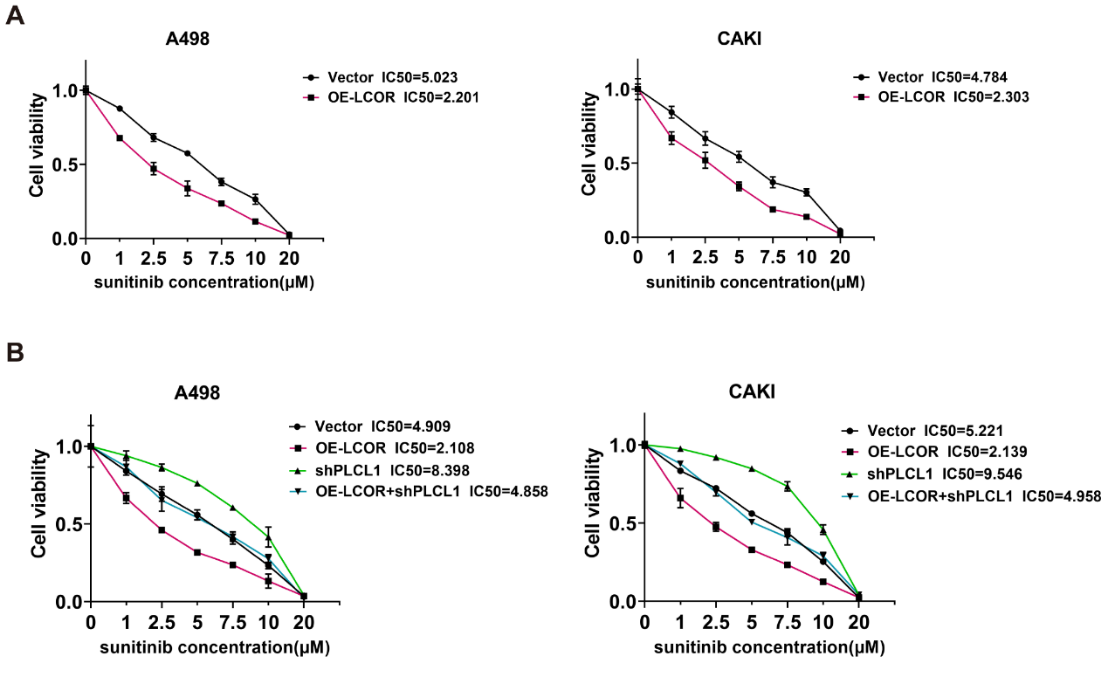

Supplementary figure 7. LCOR participated in the sensitivity of ccRCC to sunitinib. (A) Sunitinib sensitivity curves of A498 and CAKI cells with or without LCOR overexpression. **(B)** Sunitinib sensitivity assays were performed in functional compensation models.

Supplementary table 1

Sequence of primers of qPCR, siRNA

Gene	Forward	Reverse
GAPDH	GAGTCAACGGATTTGGTCGT	GACAAGCTTCCCGTTCTCAG
LCOR	GCTGACCAAGACTCACCTCT	CTTCCCTGGTTCCATCCTGT
CYP2J2	GCTTCAAAGACATGCCAGCT	GGATTGCCTGTGTGCTTTGA
ACOT11	ACCCTAGCAACCAGGTGTAC	CATGCACCACCATCTCCATG
ALOX5	GATTGTCCCCATTGCCATCC	AGAAGGTGGGTGATGGTCTG
PLCL1	CGAAGCGTTGAACTCGATGT	GAGCCATTACCTTCTGCTGC
NR2F1	CGAGGGCTGCAAAAGTTTCT	CATTCTTCCTCGCTGAACCG
RUNX1	GTGATGGCTGGCAATGATG	TGATGGCTCTGTGGTAGGTG
UCP1	GCGGATGAAACTCTACAGCG	TTGATTCCGTGGAGATGGCT
siLCOR#1	GCAGCGAAUGAUCCAACAATT	UUGUUGGAUCAUUCGCUGCTT
siLCOR#2	GCUCGAUCCCUGCAGAUUATT	UAAUCUGCAGGGAUCGAGCTT
siLCOR#3	GGAGUCUCGCACUGGUGAUTT	AUCACCAGUGCGAGACUCCTT

Supplementary table 2

Antibodies used in this study

Antibodies	Vendors	Cat#	Application
LCOR	Proteintech	14476-1-AP	WB, IHC, IP
LCOR	Santacruz	sc-398636	IF
GAPDH	ABclonal	AC002	WB
CYP2J2	ABclonal	A5805	WB
ACOT11	Sangon	D221470	WB
ALOX5	ABclonal	A2877	WB
PLCL1	Abcam	ab157200	WB
Lamin B2	ABclonal	A5001	WB
β-action	ABclonal	AC026	WB

**NR2F1**	**Proteintech**	**24573-1-AP**	**WB, IF, IP, CHIP**
**RUNX1**	**ABclonal**	**A2055**	**WB, IF**
**RUNX1**	**Proteintech**	**19555-1-AP**	**IP**
**RUNX1**	**Abcam**	**ab272456**	**CHIP**
**DDDDK-Tag**	**ABclonal**	**AE092**	**WB, IP, CHIP**
**HA**	**ABclonal**	**AE008**	**WB, IP**
**GSK-3β**	**ABclonal**	**A2081**	**WB**
**p-GSK-3β**	**ABclonal**	**AP1088**	**WB**
**β-catenin**	**ABclonal**	**A11512**	**WB**
**p-β-catenin**	**ABclonal**	**AP1315**	**WB**
**ERK**	**ABclonal**	**A16686**	**WB**
**p-ERK**	**ABclonal**	**AP0974**	**WB**
**JNK**	**ABclonal**	**A4867**	**WB**
**p-JNK**	**ABclonal**	**AP1337**	**WB**
**p38**	**ABclonal**	**A4771**	**WB**
**p-p38**	**ABclonal**	**AP1502**	**WB**
**Bcl-2**	**ABclonal**	**A25052**	**WB**
**IgG Light Chain**	**ABclonal**	**AS062**	**WB**
**UCP1**	**ABclonal**	**A7236**	**WB, IHC**
**Ki67**	**ABclonal**	**A16919**	**IHC**
